# Cancer and Its Treatment

**Published:** 1907-08-03

**Authors:** 


### CANCER AND ITS TREATMENT.

### THE PERILS OF RESEARCH.

Many of our readers will, doubtless, have seen "The Doctor's Dilemma," a play by Bernard Shaw, recently produced in London. The play contains a mixture of mocking cynicism and legitimate satire, such as Shaw has taught us to expect from his pen; cynicism in the delineation of the artist, Dubedat, satire in the sayings and doings of the six doctors who figure so conspicuously throughout the piece. With the former we are not, fortunately, concerned, but the latter may serve to point a moral to our text.

The curtain rises on Colenso Ridgeon, the famous pathologist, and discoverer of opsonins. It is the morning of his knighthood, and to him enter various of his colleagues, bringing congratulations on his honour. First comes Sir Patrick Cullen, a fine example of the old school, with a characteristic scorn for modern innovations. He would have us believe that in medicine new discoveries are never made, but that at regular intervals the old things are served up again under new names, and he chaffs Ridgeon on having hit on something which has not been "discovered" for fifty years. Then there is Cutler Walpole, the brilliant surgeon. To him all methods of treatment save the knife are as naught, and with pathology he will have no truck. Last of all Sir Ralph Blomfield Bonnington, the fashionable physician, sleek and plausible, and a very monument of complacent ignorance. With sublime assurance he talks of Ridgeon's opsonins, revealing at every word his innocence of all that concerns them, and deceiving no one, save himself, by his parrot cry of "Stimulate the Phagocytes!"

In all of this there is, of course, exaggeration; but not more than satire allows, nor so much as to obscure the likeness to the original; and the attitude of Ridgeon's friends towards his discovery reflects only too faithfully that of the majority of the medical profession towards all forms of scientific research.

### The Indifference of the Profession.

It is an attitude of indifference, sometimes also of ignorance. They neither know nor care what goes on in the laboratories, and are sadly inclined to regard those who work there as harmless enthusiasts. Towards cancer research this feeling is particularly marked, and, in addition, the most pessimistic ideas seem to prevail as to its value. Thus we are told that it has been given its chance and has failed, that it has been weighed in the balance and found wanting. We do not level this charge against every member of the profession, for there are some whose sympathetic interest comes as an oasis of green amidst the desert of neglect and discouragement, but, unfortunately, they are at present the rare exceptions.

This attitude is, we believe, based in great measure on misconceptions as to the work now being done, and the conditions under which it is carried on, and our purpose is to try to remove these misconceptions. With this object we intend to review as briefly as possible the progress made since the inauguration of what may be called the new era of cancer research, that is to say since the establishment of special laboratories devoted to this purpose. There are now in London and elsewhere several laboratories of this nature, but they are of quite recent development, and have only come into being within the last ten years. A great deal of work in connection with cancer had, of course, been done before this time, but it was for the most part the work of independent observers, who made no attempt to co-ordinate or compare their results, which did not, therefore, carry their full value. Moreover, this early work was almost entirely concerned with the histological and clinical aspects of the disease, and so thoroughly had these been investigated that research seemed to be faced by a blank wall, and further progress in this direction appeared impossible. On the establishment of organised laboratories the situation was completely changed. Combination of efforts and ideas became possible for the first time, and experimental methods of investigation were introduced, such as have given the most brilliant results in bacteriology and tropical medicine.

### The Work Accomplished.

We shall only refer here to the work of the last ten years, and in estimating the value of this it must be borne in mind that ten years is a very short space in the calendar of research. The physician or surgeon having, as a rule, no experience of laboratory work, does not realise the length of time required to yield even a small result; still less does he realise how often the labour of weeks and months may lead to no result whatsoever, so that it is necessary to hark back and try some fresh line of attack. He is, therefore, too ready to give free rein to his hopes and expectations, and when these are not at once fulfilled he is bitterly disappointed.

In the first place we may point to the great advance made in the statistical study of cancer. The work of earlier years has been collected and classified, and, in addition, exhaustive and systematic inquiries have been made as to the occurrence of cancer not only in civilised peoples, but also in the more savage races of mankind, and in the other branches of the animal kingdom. An enormous mass of facts has thus been accumulated, and, when these have been carefully sifted and examined, it will be possible to give accurate data as to the biological and geographical distribution, the mortality figure, and the sex-and-age-incidence of cancer, and to settle once and for all the vexed questions of whether it depends in any way on either heredity or contagion. Already it has been shown that cancer is not confined to the human race, but also attacks animals, birds, and fishes; and we are justified in assuming that further inquiry will prove that the entire vertebrate (and probably the invertebrate) kingdom shares with man the burden of this disease. The problem consequently assumes a much greater significance, and bears the widest biological application. Moreover, it is now evident that cancer does not depend on any of those peculiar features of environment which go to make up civilisation, but turns on some common factor equally affecting the white man, the savage, and the lower animals.

Even greater progress has ensued on the introduction of experimental methods of research. It has been found that certain cancerous tumours occurring spontaneously in mice can be artificially propagated amongst individuals of the same species, and in this way growth can be continued through many generations. Further, it is possible by previously inoculating the mouse with the blood of a healthy animal, and by certain other measures, to prevent the development of a tumour, but not to check its growth when this has once appeared. These experiments afford unequalled opportunities of studying with great exactitude the changes occurring in a tumour from the time of its first appearance until it reaches a size equal to, or even greater than, that of the animal which carries it. They have also shed light on the relations holding between the cancer and the surrounding tissues; for it has been shown that the cells of these propagated tumours are derived entirely from the cells introduced at the time of inoculation, and that the only reaction on the part of the animal inoculated lies in the formation of a connective-tissue stroma, an observation bearing directly on the hypothesis that cancer is of infective origin, as will be hereafter shown.

### The Cytology of Cancer.

This leads us naturally to the mention of recent research into the cytological aspect of malignant growth. Great interest was aroused a short time ago by the statement that the cells of a cancer, instead of following the usual somatic mode of division, which is almost universal throughout the body, followed the heterotype mode, hitherto regarded as peculiar to the reproductive cells. There was also described a cellular union or "conjugation" between the cancer cells and the leucocytes, and it was thought that this might explain the unique power of almost unlimited proliferation which malignant tissues possess. These statements have subsequently been modified; heterotype mitoses have been seen in tissues other than malignant, and doubt has been thrown on the occurrence of "conjugation." Nevertheless the increased attention directed to cytology is likely to be of great service, since it seems more and more probable that the series of phenomena, which we term cancer, depend rather on some peculiar tendency inherent in the cell than on any extrinsic influences.

### The Infective Theory.

Within the past few years a fresh stimulus has been given to the old idea that cancer may be of infective origin. The concensus of opinion at the present day is against any such explanation, but there are a few pathologists—and their judgment demands respect—who persistently maintain that the disease is due to a parasite. A micro-organism can undoubtedly be isolated from the interior of very many malignant tumours, but that this organism is the cause of the tumour has not yet been proved. Most of the clinical and pathological features of cancer certainly do not bear out such a supposition, and the fact that the cells of propagated tumours in mice are derived entirely from the few cells introduced at the time of inoculation does not coincide with experience which teaches that the reaction of healthy tissues to an infective agent is a much more definite process than the mere formation of a connective tissue stroma. We must therefore regard this question as at present unsettled, at the same time hoping that a definite conclusion will soon be reached.

### X-Ray Treatment.

Equal uncertainty exists with regard to the value of $x$-rays in the treatment of cancer. It is beyond dispute that in certain cases of carcinoma and rodent ulcer these rays have a marked influence in checking growth and promoting the healing of ulcers; whereas in other cases, apparently quite similar, they seem to have no effect whatever. It is, therefore, impossible to predict what is likely to be the result of treatment in any particular instance. This state of affairs, unsatisfactory alike to doctor and patient, is no doubt due to our ignorance as to the nature and constitution of these rays, and we may reasonably expect that further research will greatly enlarge the scope of this treatment.

There is another consideration of great interest and importance attaching to the $x$-rays, namely, the power they sometimes show of causing a malignant ulceration of any part of the skin constantly exposed to their action. This action is all the more remarkable in that it is the exact reverse of the curative effect previously mentioned, and also because it affords one of the very rare instances where the occurrence of malignant disease can be traced to some definite exciting cause.

In this brief summary we have attempted merely to touch on the salient points of progress; but we hope that enough has been said to show the pressing need for further research, and to claim for it the utmost encouragement and support.

It still remains for us to refer to yet another purpose, fulfilled by these laboratories, namely that of testing and examining the vaunted "cancer-cures" which are so numerous at the present day. The name of these is legion. They come from all parts of the world, and are usually sent by people having no scientific knowledge. But to each one that offers a semblance of a chance of success a thorough trial is given. It may be objected that such a use for research laboratories was never in the minds of those who founded them, and this no doubt is true. But the whole question of cancer stands to-day in such a peculiar position and has acquired such notoriety, that some control of this nature is a necessity in the public interest.

### The Trypsin Treatment.

One of these methods of treatment has come very prominently into notice of late, namely, the treatment of cancer by certain pancreatic enzymes, or, as it is popularly called, the "trypsin treatment." With great reluctance do we refer to this. More than enough has already been said, and much bad feeling has been aroused, but our readers may wish to know how the controversy stands at present.

This treatment differs from the majority of so-called "cancer-cures" in that it was brought forward by a distinguished embryologist, Dr. Beard, as the logical outcome of his researches; it therefore demanded careful attention and consideration. There is no need to enter into the hypotheses on which the treatment depends; suffice it to say that its author claimed for it the power to cure cancer. This claim the medical profession have refused to concede, on the grounds that there is no sufficient evidence to establish it. In consequence the supporters of trypsin have invoked the aid of the lay press in the conduct of a vigorous crusade on its behalf, and this has led to the present deplorable state of affairs. Within the past few weeks two important pronouncements as to the value of the treatment have been made; the one in the sixth report of the Cancer Research Laboratories of the Middlesex Hospital, giving the results of the treatment in a series of eleven cases; the other in the fifth annual report of the Imperial Cancer Research Fund, dealing with the attempts to check the growth of cancer in mice by this means. In either case the verdict is entirely unfavourable. Dr. Beard, however, refuses to accept these results, and states that the treatment was not carried out according to his instructions.

We hold no brief against the "trypsin-treatment," but we wish to state with all possible emphasis that to our belief there is at present no proof that the long-expected cure for cancer has been found. We would also suggest to Dr. Beard that he would be well-advised to abandon the acrimonious and abusive tone which he now employs against those who are unable to agree with him in this matter; such conduct is neither convincing nor mannerly, and is as little likely to win over his opponents as to excite the sympathy of his friends.

### Lay Advertising.

One of the most deplorable features of the situation has been the attitude of certain lay journals, who have entered on the discussion with a scarcely commendable enthusiasm. It is difficult to believe that their action has been entirely disinterested or that they can fail to realise the immense amount of harm caused by giving publicity to this and other untested remedies.

The layman is totally unable to gauge the effect of treatment in cases of cancer. He is told that as the result of certain procedure a cancerous tumour has ceased to grow, or has been diminished in size. Forthwith his thoughts follow his desires to the conclusion that a cure has been effected. But he does not know that years must elapse before a patient can be pronounced free from cancer; he does not know that almost any form of treatment when first applied will lead to a marked improvement of symptoms, only to be followed, after a longer or shorter interval, by a relapse to the old condition; he does not know how often mistakes in diagnosis are made, and tumours pronounced to be malignant which are in reality innocent. Consequently before any treatment can be stated to have a definite curative value, its effects must be carefully watched for a long period of time by an experienced observer, and to make such a statement as the result of a few months' trial shows the most culpable ignorance and neglect.

Unfortunately the victims of cancer are credulous and sanguine in the extreme, and only too willing to try any remedy which they may read of in the newspapers or hear of from their friends. By so doing they spend money which they can often ill afford, and almost invariably bring bitter disappointment on themselves and those around them. Moreover, they waste the precious time, already too short, during which surgery may give them a fair hope of recovery; for we must face the fact, however unpleasant it be, that the best if not the only chance of cure lies at present with surgery.

### What of the Future.

And, now, what are the prospects for the future? We have emphasised the value of experimental methods of investigation, we have pointed to the importance of organised laboratories in the correlation of results, and we have every confidence that these two new factors in research will lead us slowly but surely to the goal of our ambition, the discovery of a cure for cancer. It may seem idle to speculate on the probable nature of such a cure, which as yet lies hidden in the obscurity of the future, but recent work has, perhaps, thrown some light on the direction whence we may expect it to appear. According to modern conceptions it is necessary, in order to eradicate a cancer, to destroy or remove every cell having the perverted tendency of unlimited growth; if this be not done, the cancer will re-appear. So slight is the difference between the individual "malignant" cell and its normal prototype that it is almost impossible to distinguish the one from the other by microscopical or chemical tests, and it seems incredible that any drug, applied either locally or internally, can draw this distinction with such delicacy as to destroy the cancer but leave intact the healthy tissues. We require rather some agent having a specific and selective action on the cancer cells, some substance of the nature of an "anti-body," manufactured primarily by the living tissues, and not by the hand of man. If this supposition be correct the cure for cancer will lie in that wide region of serum-therapy, whose confines have only just been explored.

### The Two Forces That Retard Progress.

In the meantime cancer investigation has to contend against two opposing influences, the indifference of the profession, and the unwelcome attentions of the laity. These both tend to create a spirit of dissatisfaction with research, and thus progress is hampered and restricted. We can scarcely hope for a reversal of these attitudes, but we may with justice demand for this work greater consideration at the hands of the profession. Let them remember that the high road to success lies through many a failure, and that we can invoke no genius of the lamp to point the way to the promised land. The threads of success seem at length to lie in our hands, but only by unflagging determination and tireless effort can they be interwoven to form the fabric which all desire so anxiously to see.

